# Feasibility of Telemedicine in the Management Strategy of Patients With Lymphoma Amid the COVID-19 Pandemic in Spain: Prospective Observational Study

**DOI:** 10.2196/34128

**Published:** 2023-11-17

**Authors:** Raul Cordoba, Alberto Lopez-Garcia, Daniel Morillo, Maria-Angeles Perez-Saenz, Elham Askari, Rosa Elena Prieto, Eva Castillo Bazan, Pilar Llamas Sillero, Antonio Herrero Gonzalez, Jorge Short Apellaniz, Marta del Olmo, Javier Arcos

**Affiliations:** 1 Department of Hematology, Clinical and Organizational Innovation Unit (UICO) Fundacion Jimenez Diaz University Hospital Health Research Institute IIS-FJD Madrid Spain; 2 Department of Hematology Fundacion Jimenez Diaz University Hospital Health Research Institute IIS-FJD Madrid Spain; 3 Department of Pharmacy Fundacion Jimenez Diaz University Hospital Health Research Institute IIS-FJD Madrid Spain; 4 Department of Systems and Information Technology Fundacion Jimenez Diaz University Hospital Health Research Institute IIS-FJD Madrid Spain; 5 Clinical and Organizational Innovation Unit (UICO) Fundacion Jimenez Diaz University Hospital Health Research Institute IIS-FJD Madrid Spain

**Keywords:** telemedicine, lymphoma, COVID-19, cancer, telehealth, risk factor, patient portal, electronic health record, EHR

## Abstract

**Background:**

On March 14, 2020, a state of alarm was declared in Spain due to the spread of SARS-CoV-2. Beyond this date, COVID-19 in the country changed the practice of oncologic care.

**Objective:**

Since recurrent hospital visits were a potential risk factor for contagion, the aim of this prospective observational study was to analyze the consequences of the COVID-19 pandemic in the health care of patients with lymphoma.

**Methods:**

All data were obtained from the electronic medical record. Variables such as age, sex, reason of the visit, use of the patient portal, changes in management, enrollment in clinical trials, and COVID-19 infection were recorded.

**Results:**

In all, 290 patients visited the lymphoma clinic, totaling 437 appointments. The median age was 66 (range 18-94) years, and 157 (54.1%) patients were male. Of them, 214 (73.8%) patients had only 1 visit to the clinic. Only 23 (7.9%) patients did not have access to the patient portal. Amid the COVID-19 pandemic, 78 (26.9%) patients remained in active treatment, 35 (12.1%) experienced delays in their treatments, and 6 (2.1%) experienced treatment discontinuation. During the follow-up, only 7 (2.4%) patients had a COVID-19 infection (6 cases with confirmed polymerase chain reaction test and 1 case with clinical suspicion). Despite the implementation of telemedicine strategies to avoid visits to the hospital, 66 (22.8%) patients had in-person visits at the lymphoma clinic. Patients who attended in-person consultations were younger than those who preferred telemedicine consultations (62 vs 66 years; *P*=.10) and had less use of the patient portal (17/224, 7.6% vs 6/66, 9%; *P*=.10), although these differences did not reach statistical significance. Patients who attended in-person visits were more likely to have had only 1 visit to the hospital (29/66, 43.9% vs 185/224, 82.6%; *P*<.001). Regarding the reason of in-person consultations, more patients were on active treatment in comparison to those using telemedicine resources (37/66, 56.1% vs 42/224, 18.3%; *P*<.001). Patients with a preference for telemedicine strategies had more surveillance visits (147/224, 65.6% vs 24/66, 36.4%; *P*<.001). Regarding treatment modifications, more treatment delays (29/224, 12.9% vs 6/66, 9.1%; *P*=.10) and more definite treatment discontinuations (6/224, 2.7% vs 0/66, 0%; *P*=.10) were seen in patients using telemedicine resources when compared to patients attending in-person visits, although these differences did not reach statistical significance. Regarding the type of therapy, patients attending in-person visits were more likely to receive an intravenous treatment rather than those using telemedicine (23/66, 62.2% vs 17/224, 40.5%; *P*<.001).

**Conclusions:**

Telemedicine such as patient portals are feasible strategies in the management of patients with lymphoma during the COVID-19 pandemic, with a reduction of in-person visits to the hospital and a very low contagion rate.

## Introduction

On March 14, 2020, a state of alarm and lockdown were declared in Spain due to the spread of SARS-CoV-2. Beyond this date, COVID-19 in the country changed the practice of oncologic care, which also affected our comprehensive cancer center in Madrid. At the Oncohealth Institute, the oncologic institute for the Fundacion Jimenez Diaz University Hospital, the specialists who manage adult patients with cancer rapidly adjusted clinical practices to mitigate the potential risks of COVID-19 in patients with cancer. The new general management decisions and modifications adopted were broadly applicable to patients with solid tumors and patients with hematologic malignancies.

As of May 10, 2020, the virus had affected more than 4,000,000 individuals and resulted in almost 300,000 deaths worldwide [1]. In Spain, 269,520 COVID-19 cases confirmed by polymerase chain reaction (PCR) tests and 26,920 deaths were reported by health authorities, thus ranking the country as fifth in the world with the highest mortality rate. Risk factors for COVID-19 severity and death include older age, along with comorbidities such as diabetes, hypertension, or cardiac disease, with a mortality rate as high as 15% in older adults and patients with comorbidities [2].

In the first reports in China, patients with cancer seemed to have a significantly higher incidence of severe events, including intensive care unit admission, need of assisted ventilation, or death after contracting the virus, with a rate of 39% versus 8% in patients without cancer [3].

Regarding patients with hematological malignancies, the first evidence reported 13 cases in a cohort of 128 hospitalized patients with hematological malignancies, with no significant differences in baseline covariates between patients developing or not developing COVID-19. Cases with concurrent hematologic neoplasm and COVID-19 had a more severe manifestation of the disease, including those with acute respiratory distress syndrome (ARDS) having a higher mortality rate [4].

In a recent study conducted at a single center in Madrid, one of the outbreak epicenter in Spain, 34 cases with hematological malignancies were reported. The mortality rate was 32.35% with a median follow-up of 26 days, and the overall survival rate was 67%. In the subgroup analysis for hematological malignancy subtype, no deaths were observed in the group of patients with lymphoid malignancies, including patients with non-Hodgkin lymphoma, Hodgkin lymphoma, and chronic lymphocytic leukemia. Only hematologic status (initial diagnosis, relapsed or refractory disease, stable disease, remission without therapy, or watch-and-wait strategy), Eastern Cooperative Oncology Group performance status, and ARDS were independent variables for overall survival [5].

Since hospital admission and recurrent hospital visits inherent to the management of patients with cancer are potential risk factors for SARS-CoV-2 infection [6], there was no specific information for patients with lymphoma.

Before the COVID-19 pandemic, telehealth was starting to be explored with the use of electronic services and devices to support a broad range of remote services, such as patient care and monitoring. When thinking about the implementation of telemedicine strategies in patient care, the 6 domains of care quality defined by the Institute of Medicine should be addressed: safe, effective, patient-centered, timely, efficient, and equitable [7]. Stakeholders may see telehealth as a disruptive technology that appears to threaten traditional health care delivery but that also has the potential to transform the way of providing health services by reducing costs and increasing quality and patient satisfaction.

Telehealth technology is an excellent solution to solve problems of health care delivery, which appears to be the case in the COVID-19 outbreak. However, this technology may fail during large-scale implementation. In a recent systematic review, the applications of the existing business models were reported in several studies related to different types of services, namely, telemonitoring, telemedicine, mobile health, telehealth, assisted living technologies, and sensor-based systems [8]. After the lockdown and travel restrictions in the country, different strategies were analyzed to keep on providing health care services to patients, at least to those more vulnerable from experiencing a severe COVID-19 infection.

When thinking about different telemedicine strategies, two types of teleconsultation were distinguished: (1) asynchronous teleconsultation for monitoring and delivering feedback via email and phone calls, automated messaging systems, or other equipment without face-to-face contact such as questionnaires sent through a patient portal platform; and (2) synchronous teleconsultation that involves real-time, face-to-face contact via videoconferencing equipment to connect caregivers and one or more patients simultaneously [9].

The aims of this prospective, nonrandomized study were to analyze the consequences of the COVID-19 pandemic in the health care of patients with cancer at the lymphoma clinic, to describe the telemedicine strategies explored in our institution and how these strategies evolved during the COVID-19 pandemic, and to see the impact of the telemedicine in the behavior of patients with lymphoma to avoid visits to the hospital, albeit maintaining close monitoring and follow-up, with a special focus on older adults.

## Methods

### Patients

From March 16 to May 8, 2020, data from all appointments at the lymphoma clinic at the Oncohealth Institute of Fundacion University Hospital in Madrid (Spain) were collected. All patients with scheduled appointments at the lymphoma clinic were offered to participate in the study with no exclusion criteria. Patients must be registered in the hospital patient portal for further communication. Patients without smartphones or access to the internet were recruited with the support of family members or caregivers. All data were obtained from the electronic medical record. Variables such as age, sex, reason of the visit (eg, surveillance and ongoing therapy), transformation of a standard visit into telemedicine, use of the patient portal, changes in management, enrollment in clinical trials, and COVID-19 infection were recorded.

### Ethics Approval

This prospective, nonrandomized study was approved by the institutional ethics committee (PIC_FJD_213/19) and was performed in accordance with the ethical standards as laid down in the 1964 Declaration of Helsinki and its later amendments.

### Telemedicine Strategies

From the beginning of the state of alarm in the country due to the COVID-19 pandemic, all standard appointments were cancelled to avoid patients visiting the hospital and thus reducing their risk of SARS-CoV-2 infection. In-person evaluation, phone consultations, and communication through the patient portal were the 3 different proposed strategies to modify the logistics of health care. Prior to enrollment, all patients were registered in the hospital patient portal and receive instructions about how to use it. Two specific questionnaires with disease-related and COVID-19–related questions were uploaded to the patient portal, and they were sent 48-72 hours prior to the scheduled appointment. If the questionnaires were not submitted, a case manager conducted a phone call.

### Statistical Analysis

Means between groups were compared using independent group 2-tailed *t* test and Mann-Whitney *U* test for normally and abnormally distributed data, respectively. Proportions for categorical variables were compared by chi-square test or Fisher test when appropriate. Cox proportional hazards models were constructed based on univariate and multivariate analysis results, and variables for multivariate models were chosen based on clinical relevance.

## Results

In the first 8 weeks of the state of alarm and travel restrictions, 290 patients visited the lymphoma clinic, totaling 437 appointments. Baseline patient characteristics are shown in Table 1. The median age was 66 (range 18-94) years, and 157 (54.1%) patients were male. According to age subgroups, 112 (38.6%) patients were younger than 60 years, 69 (23.8%) were between 61-70 years, 64 (22.1%) were between 71-80 years, 39 (13.4%) were between 81-90 years, and 6 (2.1%) were older than 90 years. Among all patients included in the study, 214 (73.8%) had only 1 visit to the clinic, whereas the remaining 76 (26.2%) were frequent users with more than 1 visit, with a median of 2 (range 2-8) visits. Only 23 (7.9%) patients did not have access to the patient portal, so communication with them could only be conducted in person or by phone calls. The reasons for not having access to the patient portal were language barrier (n=5, 1.7%), no access to the internet (n=17, 5.9%), and reluctance to use (n=1, 0.3%). Age by itself was not a barrier. Older adults were supported by family members and caregivers to manage the patient portal tool. Amid the COVID-19 pandemic, 78 (26.9%) patients remained in active treatment, 35 (12.1%) experienced delays in their treatments (in most of the cases, the deferred therapy was rituximab maintenance in patients with non-Hodgkin lymphoma in complete remission), and 6 (2.1%) experienced treatment discontinuation (4 [1.4%] patients because of personal preference, 1 [0.3%] because of toxicity, and only 1 [0.3%] died with concomitant COVID-19 and lymphoma). The remaining 171 (59%) patients were in active surveillance. Of note, 27 (9.3%) patients were participating in clinical trials, although enrollment was done prior to the pandemic. Of them, only 3 (1%) patients did not complete the trial procedures: 1 (0.3%) died due to COVID-19 pneumonia, 1 (0.3%) preferred to miss one dose of treatment, and 1 (0.3%) withdrew consent before starting therapy. During the follow-up, only 7 (2.4%) patients had a COVID-19 infection (6 cases with confirmed PCR test and 1 case with clinical suspicion). In all, 3 (1%) cases had contact with a known familiar case, and in the remaining 4 (1.4%) patients, the origin of infection was unknown. There was only 1 (0.3%) fatal case in a patient with follicular lymphoma receiving rituximab monotherapy. The other 6 (2.1%) cases required hospital admission—with none of them requiring intense care unit admission—and were discharged without sequelae.

**Table 1:** Patient characteristics.

Characteristic		All patients (N=290)	Patients with in-person visits (n=66)	Patients with telemedicine visits (n=224)	*P* value	
Age (years), median (range)		66 (18-94)	62 (24-91)	66 (18-94)	.10	
Sex, male, n (%)^a^		157 (54.1)	35 (53)	122 (54.5)		
**Number of visits, n (%)^a^**						
	1 visit	214 (73.8)	29 (43.9)	185 (82.6)	<.001	
Access to the patient portal, n (%)^a^		267 (92.1)	60 (90.9)	207 (92.4)	.10	
**Reason of the visit, n (%)^a^**						
	Surveillance	171 (56)	24 (36.4)	147 (65.6)	<.001	
	Active treatment	78 (26.9)	37 (56.1)	42 (18.3)	<.001	
	Treatment delay	35 (12.1)	6 (9.1)	29 (12.9)	.10	
	Discontinuation	6 (2.1)	0 (0)	6 (2.7)	.10	
**Type of therapy, n (%)^b^**						
	Oral	36 (46.2)	12 (32.4)	24 (58.5)		
	IV^c^	40 (51.3)	23 (62.2)	17 (40.5)	<.001	
	Subcutaneous	2 (2.6)	2 (5.4)	0 (0)		
Patients in clinical trials, n (%)^a^		27 (9.3)	15 (22.7)	12 (5.4)		
COVID-19 infection, n (%)^a^		7 (2.4)	4 (6.1)	3 (1.3)		

^a^Percentages were obtained in reference to the number of visits (all patients: n=290; patients with in-person visits: n=66; patients with telemedicine visits: n=224).

^b^Percentages were obtained in reference to the total number of patients on active therapy (all patients: n=78; patients with in-person visits: n=37; patients with telemedicine visits: n=41).

^c^IV: intravenous.

**Figure 1:**
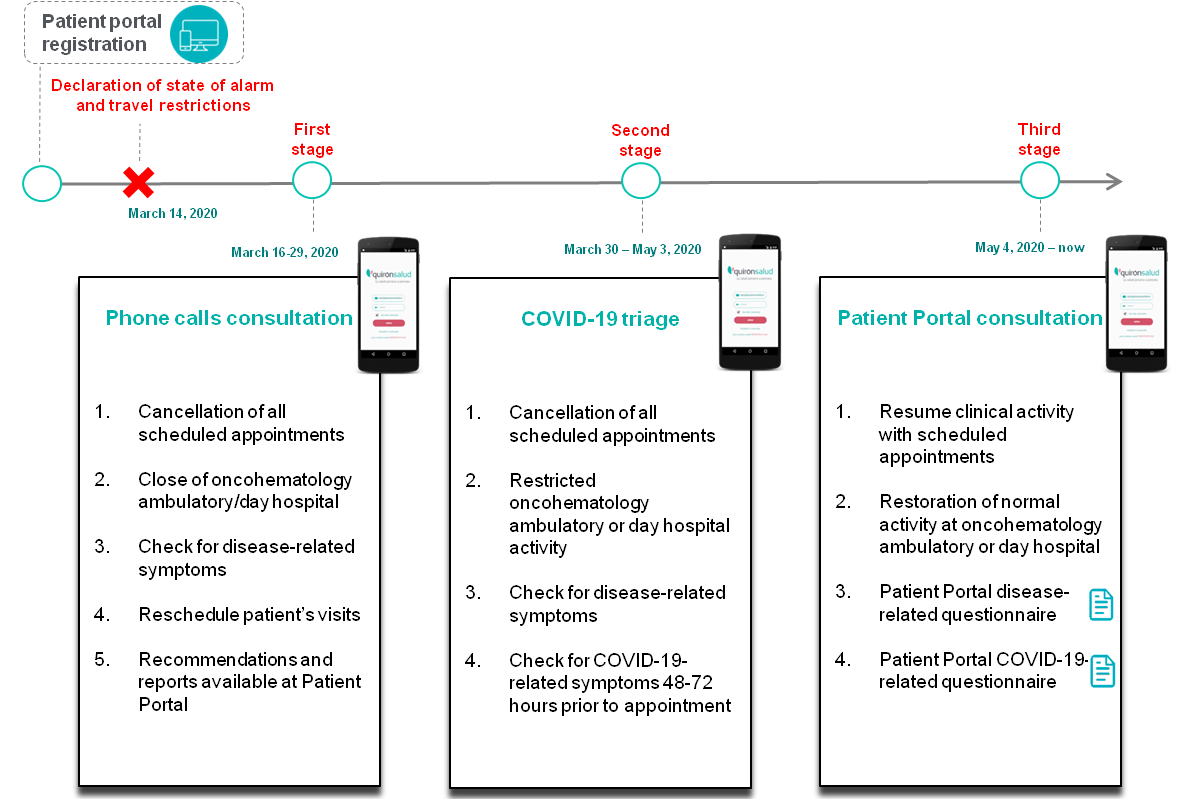
Telemedicine strategies amidst the state of alarm and confinement due to COVID-19 outbreaak and evolution during the pandemic.

Telemedicine resources were offered to all patients amid the state of alarm and travel restrictions. These resources evolved during the pandemic (Figure 1). In the first period, from March 16 to March 29, 2020, all patients with a non–in-person visit were contacted by phone to give assessment results if the blood tests or imaging reports were done before the lockdown or to give advice about what to do (arrange an in-person visit to the hospital or delay the consultation until pandemic was resolved or under control). Patients with access to their patient portal got access to a report about their consultation. In the second period, from March 30 to May 3, 2020, with the peak of new cases and deaths, a phone triage was done at least 48-72 hours before the appointment to check about symptoms, if they have been in contact with a known COVID-19–infected person, and the place of residence (home, long-term care facilities, or nursing home). Patients with a positive triage were referred to the COVID-19–designated areas for SARS-CoV-2 PCR testing with a nasopharynx swab and to specific blood-drawing area, day hospital, and in-patient wards. Patients with access to their patient portal also got access to a report about their consultation. In the third period, from May 4, 2020 until now, all patients were invited to have a remote visit through the patient portal without phone calls. Patients received a questionnaire through their patient portal screening for lymphoma-related symptoms. Blood tests and imaging procedures were scheduled, and a final report with all the results were created in the electronic medical record and with access through the patient portal.

Despite the implementation of telemedicine strategies to avoid visits to the hospital, 66 (22.8%) patients had in-person visits at the lymphoma clinic. Patients who attended in-person consultations were younger than those who preferred telemedicine consultations (62 vs 66 years; *P*=.10) and had less use of the patient portal (17/224, 7.6% vs 6/66, 9.1%; *P*=.10), although these differences did not reach statistical significance. There were no differences among men and women. Patients who attended in-person visits were more likely to have had only 1 visit to the hospital (29/66, 43.9% vs 185/224, 82.6%; *P*<.001). Regarding the reason of in-person consultations, more patients were on active treatment in comparison to those using telemedicine resources (37/66, 56.1% vs 42/224, 18.3%; *P*<.001). Patients with a preference for telemedicine strategies had more surveillance visits (147/224, 65.6% vs 24/66, 36.4%; *P*<.001). Regarding treatment modifications, more treatment delays (29/224, 12.9% vs 6/66, 9.1%; *P*=.10) and more definite treatment discontinuations (6/224, 2.7% vs 0/66, 0%; *P*=.10) were seen in patients using telemedicine resources when compared to patients attending in-person visits, although these differences did not reach statistical significance. Regarding the type of therapy, patients attending in-person visits were more likely to receive an intravenous treatment rather than those using telemedicine (23/66, 62.2% vs 17/224, 40.5%; *P*<.001). Clinical trial activity in already recruited patients was not altered. Of the 27 patients on active treatment, 15 patients attended in-person visits and 12 patients attended using telemedicine resources, with the sponsor’s agreement.

Regarding frequent users, patient characteristics are shown in Table 2. Both groups had a similar median age and male-to-female ratio. Frequent users had more in-person visits (37/76, 48.7% vs 29/214, 13.6%; *P*<.001), were receiving more active treatment (49/76, 64.5% vs 30/214, 14%, *P*<.001), and less access to the patient portal (9/214, 4.2% vs 4/76, 5.3%; *P*=.10)—although without statistical significance.

**Table 2:** Patient characteristics of those with a single visit to the hospital versus frequent users.

Characteristic			Patients with a single visit to the hospital (n=214)		Frequent users (n=76)		*P* value
Age (years), median (range)			66 (18-94)		65 (25-93)		
Sex, male, n (%)			110 (51.4)		47 (61.8)		
**Type of visit, n (%)**							
	In-person	29 (13.6)		37 (48.7)		<.001	
	Telemedicine	185 (86.4)		39 (51.3)			
**Reason of the visit, n (%)**							
	Surveillance	152 (71)		18 (23.7)			
	Active treatment	30 (14)		49 (64.5)		<.001	
	Treatment delay	27 (12.6)		8 (10.5)			
	Discontinuation	5 (2.3)		1 (1.3)			
Access to the patient portal, n (%)			205 (95.8)		72 (94.7)		.10

## Discussion

### Principal Findings

Patients with hematologic malignancies appear to be a very vulnerable population to COVID-19 infection. In a French study [10], 25 out of 48 patients with suspicion of COVID-19 eventually had a confirmed positive test. Patients with a hematologic malignancy were at a higher risk of developing a severe form of COVID-19 with ARDS, requiring mechanical ventilation, compared to the general population without an underlying medical condition, and this translated into a very high mortality rate with an estimate of 40% at 1 month. Only 8 out of 25 patients had a diagnosis of any type of mature lymphoid malignancies, so very scarce information was available at that time.

The first concern when talking about telemedicine is always its accessibility, at least in remote areas. In our setting, only fewer than 10% of patients did not have access to the internet, and they were not users of the patient portal provided by the institution; however, when analyzing the reasons, the principal factor was language barrier in non–Spanish-speaking patients rather than access to the internet. Foreign populations are even more vulnerable in outbreak situations. As our institution is located within the city limits of Madrid and is easily accessed by public transportation, we have seen a group of patients who preferred to attend an in-person visit despite the risk of infection only because of proximity, but the majority, following governmental instructions of travel restrictions, preferred to stay safe at home. Another obstacle seen by many physicians was the impossibility of conducting an appropriate physical examination from a distance. To overcome this issue, we implemented a questionnaire that was sent to the patients through the patient portal, specifically design for lymphoma symptoms screening. When any of the questions was answered positively, an immediate action was taken. Patient satisfaction of this questionnaire was very high, with a Net Promoter Score of 70.

Patients were divided into 3 subgroups with different recommendations following international guidelines recommendations: the first group consisted of patients with cancer in the surveillance program or on oral antineoplastic therapy; the second group consisted of patients with a recently diagnosed cancer who needed therapy with curative intent; and the third group consisted of patients in a relapse or refractory setting receiving palliative treatment [11,12]. In our study, more than one-quarter of the patients remained in active treatment, where we considered that the benefit of receiving therapy outweighs the risk of COVID-19 infection.

Clinical trial activity was not altered in patients already recruited and on active therapy. Agreement with sponsors allow us to skip in-person visits and blood drawings for postdose pharmacokinetics. The enrollment of new patients in clinical trials was reduced, and no patients were included in trials amid the pandemic.

### Comparison With Prior Work

In our study, the mortality rate was very low, with only 1 death in a patient with follicular lymphoma who was in active treatment with rituximab monotherapy. Our results are similar to those published in another Spanish study [5], where 12 patients with lymphoma and COVID-19 were recruited and no deaths occurred among this subgroup in comparison to other hematological malignancies.

To avoid hospital admission and recurrent hospital visits inherent to the management of patients with cancer, several groups have proposed telemedicine strategies to reduce the potential risk factors for SARS-CoV-2 infection [13,14]. Telemedicine may be used to support patients to minimize the number of visits and risk of exposure. Several governments imposed confinement rules, limiting all kinds of transportation and, therefore, visit to hospitals. Telemedicine and digital strategies have been adopted by many scientific societies and hospitals as being the safest, both for the patient and the medical personnel. This new way of clinical practice has made us wonder whether telemedicine is a threat to patient care in oncology and hematology or if it is an opportunity that could revolutionize our clinical practice by empowering both patients and physicians [15].

In a recent review covering the literature on telehealth during the first 6 months of the COVID-19 pandemic, there is compelling evidence to suggest that telehealth may have a significant effect on advancing health care in the future. However, the feasibility and application of telehealth in resource-limited settings and low- and middle-income countries must be established to avail its potential and transform health care for the world’s population. Given the rapidity with which telehealth is advancing, a global consensus on definitions, boundaries, protocols, monitoring, evaluation, and data privacy is urgently needed [16].

Patients from different age groups and with different health conditions benefited from remote health services. Telehealth consultation via videoconferencing was effective in delivering web-based treatment and was well accepted by patients, as it simulated in-person, face-to-face consultations. Acceptance by patients increased as a result of web-based consultation facilitators that promoted effective and convenient remote treatment. However, some patients preferred face-to-face consultations and showed resistance to web-based consultations [17]. Other telehealth consultations such as the use of patient portals may be of help in overcoming this initial reluctant to use.

There are several advantages of telemedicine in the care of patients with lymphoma. Patients living in remote areas or those with travel restrictions, as occurred in many countries amid the COVID-19 outbreak, do not need to travel a long time and can easily get access to their medical team for routine questions and answers. This is also of particular interest for patients on oral antineoplastic treatments who have to come by in person to refill prescriptions. Our Department of Pharmacy also implemented a home-delivery system to deliver the prescriptions directly into patients’ homes. Another advantage is the easier and more frequent way to check that patients were adhering to the given recommendations, by sending them checklists or questionnaires to be completed on a regular basis. In this regard, we have been using the patient portal to send questionnaires for a close monitoring of symptoms, adverse events, and quality of life. Since December 2019, we have started a surveillance program using patient-reported outcome measurement and patient-reported experience measurement in patients with hematological cancers. This program, which was implemented before the COVID-19 pandemic, helped us in the close monitoring of our patients.

### Limitations

Because of the nature of the study, the main limitation was the impossibility to compare this telemedicine strategy with the standard in-person clinical practice to determine if it is better or not. If we compare our study to other experiences worldwide, although telemedicine will not solve all challenges, it can provide rapid access to specialists who are unavailable in person. With the rapid development of the internet and smartphone apps, telemedicine has transitioned to a multimodal paradigm, offering greater possibilities and convenience. Telemedicine has prerequisites for success that include sufficient financial resources, technological infrastructure, and the overall alignment of policy makers [18].

In the future, large-scale prospective studies are needed to see how to improve and implement telemedicine and digital health strategies and whether they will soon become a new standard of care in treating patients with cancer. Further follow-up is needed to assess the impact of the pandemic and the telemedicine strategies in the outcome of our patients.

### Conclusions

With the COVID-19 outbreak, where cities experience lockdown due to fear from contagion, telemedicine has demonstrated the advantages of this new way of attending patients with cancer. This fact is even more relevant in potentially curable malignancies such as lymphomas, where limitations are clearly outweighing the setbacks. What is more important is that both physicians and patients are learning how to use these technologies and are incorporating them into their clinical practice. Older adults are not out of the reach of this digital revolution, and we have to make efforts to integrate them in the new digital era of medicine.

## Abbreviations

ARDS: acute respiratory distress syndrome
PCR: polymerase chain reaction

## Data Availability

The data sets generated during and/or analyzed during the current study are available from the corresponding author on reasonable request.
